# Formation of the initial kidney and mouth opening in larval amphioxus studied with serial blockface scanning electron microscopy (SBSEM)

**DOI:** 10.1186/s13227-018-0104-3

**Published:** 2018-06-21

**Authors:** Nicholas D. Holland

**Affiliations:** Marine Biology Research Division, Scripps Institution of Oceanography, University of California at San Diego, 9500 Gilman Drive, La Jolla, CA 92093 USA

**Keywords:** Cephalochordata, Amphioxus, Lancelet, Kidney, Mouth evolution, Serial blockface scanning electron microscopy (SBSEM)

## Abstract

**Background:**

For early larvae of amphioxus, Kaji et al. (Zool Lett 2:2, 2016) proposed that mesoderm cells are added to the rim of the forming mouth, giving it the quality of a coelomoduct without homology to the oral openings of other animals. They depended in part on non-serial transmission electron microscopic (TEM) sections and could not readily put fine structural details into a broader context. The present study of amphioxus larvae is based largely on serial blockface scanning electron microscopy (SBSEM), a technique revealing TEM-level details within an extensive anatomical volume that can be reconstructed in three dimensions.

**Results:**

In amphioxus larvae shortly before mouth formation, a population of compact mesoderm cells is present at the posterior extremity of the first left somite. As development continues, the more dorsal of these cells give rise to the initial kidney (Hatschek’s nephridium), while the more ventral cells become interposed between the ectoderm and endoderm in a localized region where the mouth will soon penetrate. SBSEM reveals that, after the mouth has opened, a majority of these mesoderm cells can still be detected, sandwiched between the ectoderm and endoderm; they are probably myoblasts destined to develop into the perioral muscles.

**Conclusions:**

SBSEM has provided the most accurate and detailed description to date of the tissues at the anterior end of amphioxus larvae. The present study supports the finding of Kaji et al. (2016) that the more dorsal of the cells in the posterior region of the first left somite give rise to the initial kidney. In contrast, the fate of the more ventral cells (called here the oral mesoderm) is less well understood. Although Kaji et al. (2016) implied that all of the oral mesoderm cells joined the rim of the forming mouth, SBSEM reveals that many of them are still present after mouth penetration. Even so, some of those cells go missing during mouth penetration and their fate is unknown. It cannot be ruled out that they were incorporated into the rim of the nascent mouth as proposed by Kaji et al. (2016). On the other hand, they might have degenerated or been shed from the larva during the morphogenetic interaction between the ectoderm and endoderm to form the mouth. The present SBSEM study, like Kaji et al. (2016), is based on static morphological data, and dynamic cell tracer experiments would be needed to decide among these possibilities.

## Background

The present study was prompted by Kaji et al. [1], who focused on the development of mesoderm cells in the posterior wall of the first left somite of amphioxus larvae. According to those authors, some of the cells in question gave rise to the initial kidney (Hatschek’s nephridium), while others were added to the rim of the forming mouth. This mesodermal intercalation was interpreted to mean that the amphioxus mouth is actually a coelomoduct and thus not homologous with the oral opening of any other animal. The oral coelomoduct scenario was based on a combination of molecular genetic data (considered in the “Discussion” section) and conventional transmission electron microscopy (TEM). The TEM sections in Ref. [1] were not collected serially and consequently were difficult to relate to the overall structure of the larva. Therefore, the present investigation of kidney formation and mouth penetration in amphioxus larvae is based primarily on serial blockface scanning electron microscopy (SBSEM), a technique permitting the detailed study of cells and tissues within the context of extensive regions of the body.

For SBSEM, a resin-embedded sample is introduced into a specimen chamber containing an ultramicrotome equipped with a diamond knife and a field emission scanning electron microscope (SEM). After the blockface is imaged by backscattered electrons, a thin section is shaved away from the specimen and discarded, thereby exposing a new surface for the next scan [2, 3]. The alternation of section removal and blockface scanning generates uninterrupted serial images superficially resembling conventional TEM, although with a somewhat lower resolution. Because of this resolution problem (exacerbated by the relatively low magnifications needed for sampling large tissue volumes), the present study was augmented by some conventional, non-serial TEM. The results described here are in general agreement with Ref. [1] about amphioxus nephrogenesis (reviewed in [4]). In contrast, no clear support was found that mesoderm cells (termed “mesovesicle” cells by Ref. [1] but designated here with the less stage-specific name of “oral mesoderm” cells) are added to the rim of the mouth. Instead, many of the cells in question can still be detected after the mouth opens and are probably myoblasts destined to differentiate into the perioral musculature. However, some of the oral mesoderm cells cannot be accounted for after mouth opening, and their fate remains unknown.

## Methods

### Raising amphioxus; somite numbering conventions and terminology

**Fig. 1:**
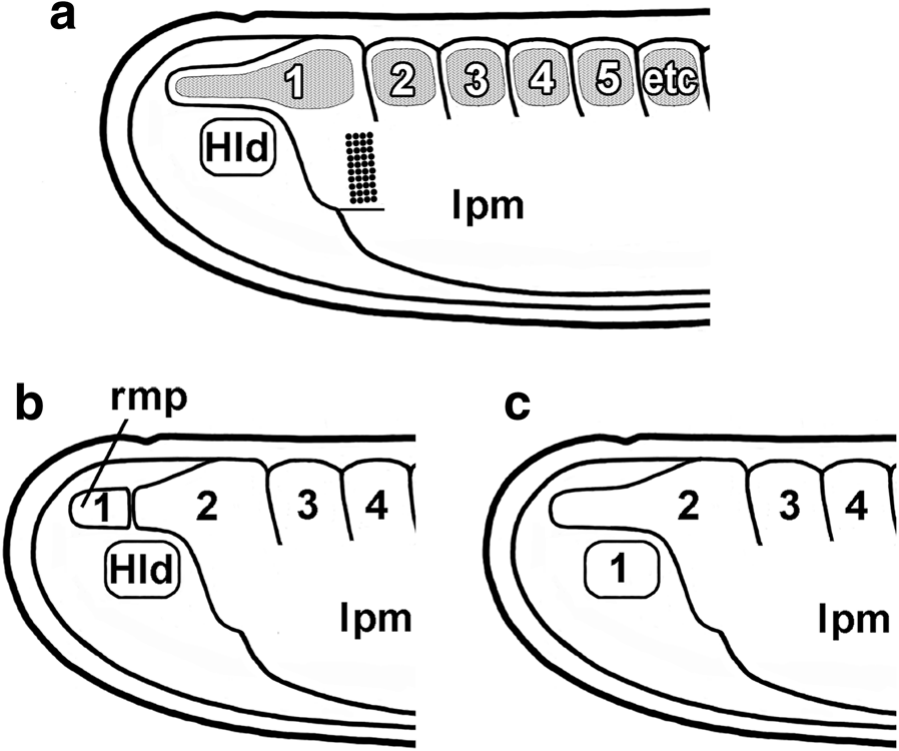
The anterior region of amphioxus larvae before mouth opening. The somites (shaded) give rise to and are continuous with the lateral plate mesoderm (lpm). Historically, amphioxus somites have been numbered in three different ways. **a** The present study follows [43] in equating somites to myotomes (shaded). **b** Alternatively, Hatschek [44] counted a rostral muscular process (rmp) as the first myotome, but later abandoned this idea [45]. **c** In the third scheme, several authors [19, 26, 37, 46] defined Hatschek’s left (Hld) and right diverticula as the first somites. Coarse stippling in **a** indicates the zone of compact mesoderm cells in the first left somite

Specimens of the Florida amphioxus, *Branchiostoma floridae*, originally collected in Tampa Bay, have been maintained in continuous breeding culture for the past decade at Scripps Institution of Oceanography in La Jolla. Males and females are fed microalgae and ripened in the laboratory at 17 °C on a photoregime of 10 h dark/14 h light [5]. At any time of year, when the temperature is elevated to 24 °C for 24 h, the animals spawn a few minutes after the onset of the next day’s dark period. Eggs are fertilized by adding sperm, and the resulting embryos are raised at 27 °C. For simplicity, all the developmental stages in this study will be called larvae (even though some alternative schemes consider stages prior to mouth opening to be embryos). The terminology used here for mesodermal structures in amphioxus (and in chordates generally) follows Ref. [6]. It is useful to keep in mind that amphioxus somites give rise to and are contiguous ventrally with a sheet of non-segmented lateral plate mesoderm. During subsequent development, this sheet splits by schizocoely into a visceral and parietal peritoneum enclosing a narrow perivisceral coelom. In addition, to make sense of the older literature on amphioxus segmentation, one needs to be aware that there have been three alternative schemes for numbering the somites (Fig. 1a–c). The one used here is illustrated in Fig. 1a.

### Optical microscopy, conventional SEM, and conventional TEM

Larvae at stages before and after mouth opening were photographed alive by differential interference contrast (DIC) optical microscopy. A more precise time course for mouth opening was determined by conventional SEM of samples of ten larvae fixed each hour between 20 and 25 h after fertilization. After fixation overnight at 4 °C in 2% glutaraldehyde–sea water, specimens were rinsed for 30 s in distilled water, dehydrated in ethanol, transferred to hexamethyldisilazane [7], and air-dried. Dried larvae were mounted left side up on double-sided adhesive tape affixed to SEM stubs, coated with iridium, and viewed in a Hitachi S4800 SEM.

For conventional TEM, six larvae were fixed every 30 min between 23 h and 25 h. Fixation was for 90 min at room temperature in 3% glutaraldehyde in 0.1 M phosphate buffer (pH 7.3) with 0.5 M sucrose [8]. Specimens were rinsed in three 5-min changes of 0.1 M phosphate buffer (pH 7.3) with 0.5 M sucrose and then post-fixed in 1% osmium tetroxide in 0.1 M phosphate buffer (pH 7.3) with 0.5 M sucrose at 4 °C for 1 h. After dehydration at room temperature in an ethanol series, larvae were transferred to propylene oxide, embedded in LX-112 resin, and oriented for cross sectioning. At each time sampled, 2-μm-thick sections cut with a glass knife were prepared alternatively with diamond cut runs of half a dozen fine sections picked up on filmed grids. The fine sections were stained in uranyl acetate and lead citrate and viewed by TEM in a Phillips CM100 microscope.

### Processing for SBSEM and three-dimensional (3-D) reconstruction

For SBSEM, a single larva was fixed at each of the following times after fertilization: 22, 23, 24, and 25 h (newly open mouth). Ancillary observations were made on six additional larvae for which SBSEM processing was terminated early when structural damage or poor orientation was detected during the procedure. The primary fixation was for 2 weeks at 4 °C in 0.15 M cacodylate buffer (pH 7.4) containing 2.5% glutaraldehyde, 2% formaldehyde, and 2 mM CaCl_2_ [9]. After primary fixation, the larvae were exposed successively to reduced osmium tetroxide, thiocarbohydrazide, osmium tetroxide, uranyl acetate, and lead aspartate under conditions in Table 1 in Ref. [10]. Following ethanol dehydration at 4 °C, the specimens were transferred through acetone and embedded in Durcupan resin. SBSEM was carried out with a 3View system (Gatan, Pleasanton, CA) installed in a Zeiss Merlin SEM. Blockfaces were scanned every 250 nm. The SBSEM image series was converted into 3-D with Reconstruct software, available gratis from http://www.bu.edu/neural/Reconstruct.html [11, 12]. In the 3-D reconstructions, most of the cells and tissues studied were depicted as continuous Boissonnat surfaces (in some instances, rendered semitransparent), although basal laminae were visualized as non-merged traces made on every third image. Finally, unless otherwise noted, transverse images are oriented as if viewed from the posterior end of the larva.

### Adjusting to the technical limitations of SBSEM

**Fig. 2:**
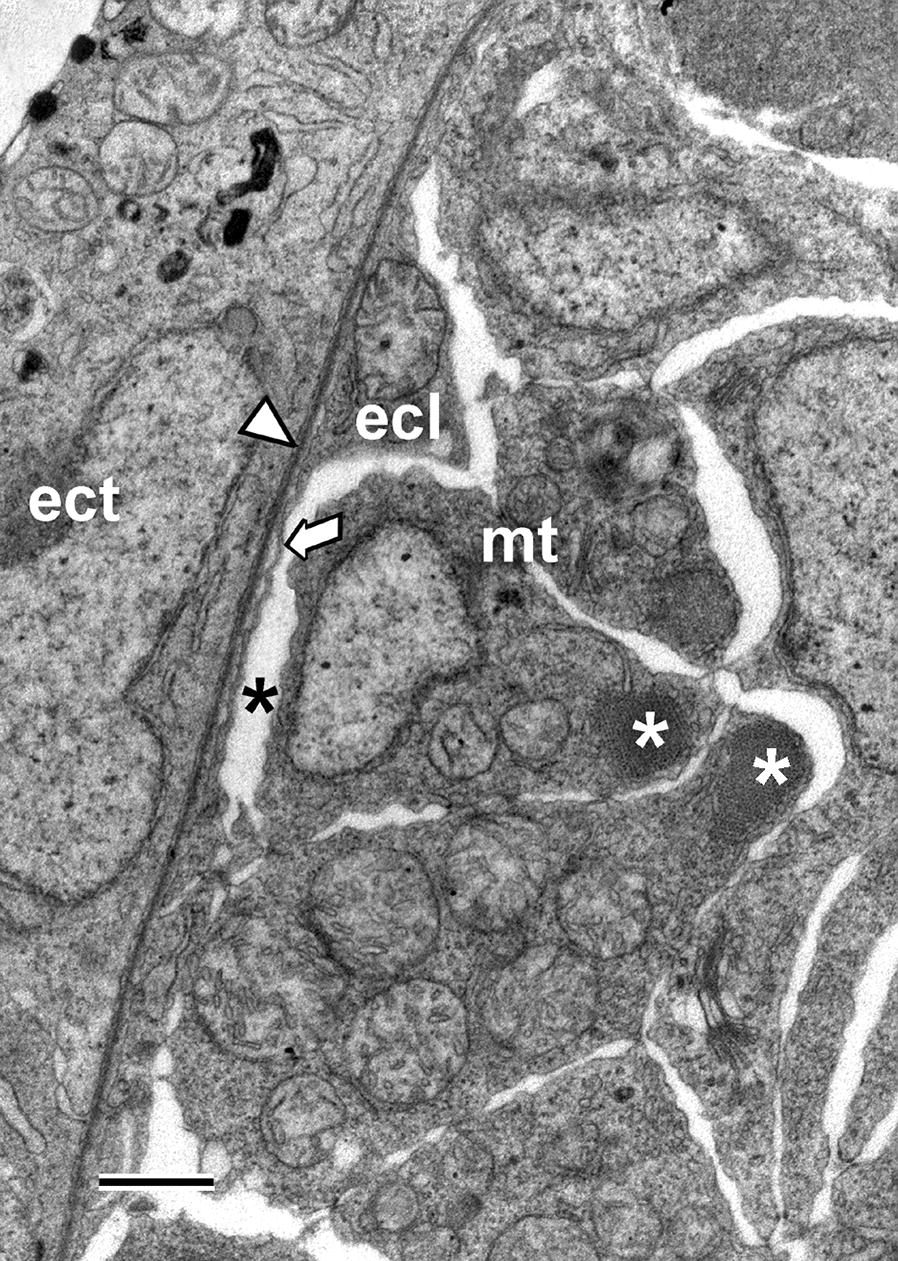
Conventional TEM of a cross section of a somite of an amphioxus larva shortly before mouth opening. The ectoderm (ect) is underlain by a basal lamina (arrowhead). The external cell layer (ecl) includes extremely thin cytoplasmic extensions (arrow) and is separated by an inconspicuous myocoel (black asterisk) from the myotome (mt). The myoblasts of the myotome contain myofilament bundles (white asterisks). Scale bar = 1 μm

The presentation of the results takes into account the relatively low resolution of SBSEM. The technique, when used at the low magnifications needed for the present study, cannot reliably detect very thin cytoplasmic extensions of cells and narrow lumina that would be visible in conventional TEM (examples of such features are shown in Fig. 2). Thus, in the present study, whereas relatively compact cells could be reliably visualized in their entirety, cells with extensive cytoplasmic extensions could not. For these latter cells, only the nuclei were reconstructed to give some idea of the overall distribution and density of the cells within a tissue. Very narrow lumina, like the one within the early Hatschek’s nephridium, could not be adequately visualized, but, unexpectedly, even the more voluminous lumen of the nascent pharynx was sometimes shrunken. The prevention of such artifactual shrinkage of spaces by adjusting conditions during preparation for SBSEM was not attempted because the transfer of specimens through the six sequential fixation and post-fixation solutions greatly complicates identification and adjustment of the critical variable(s).

## Results

### Time course for the opening of the larval mouth

**Fig. 3:**
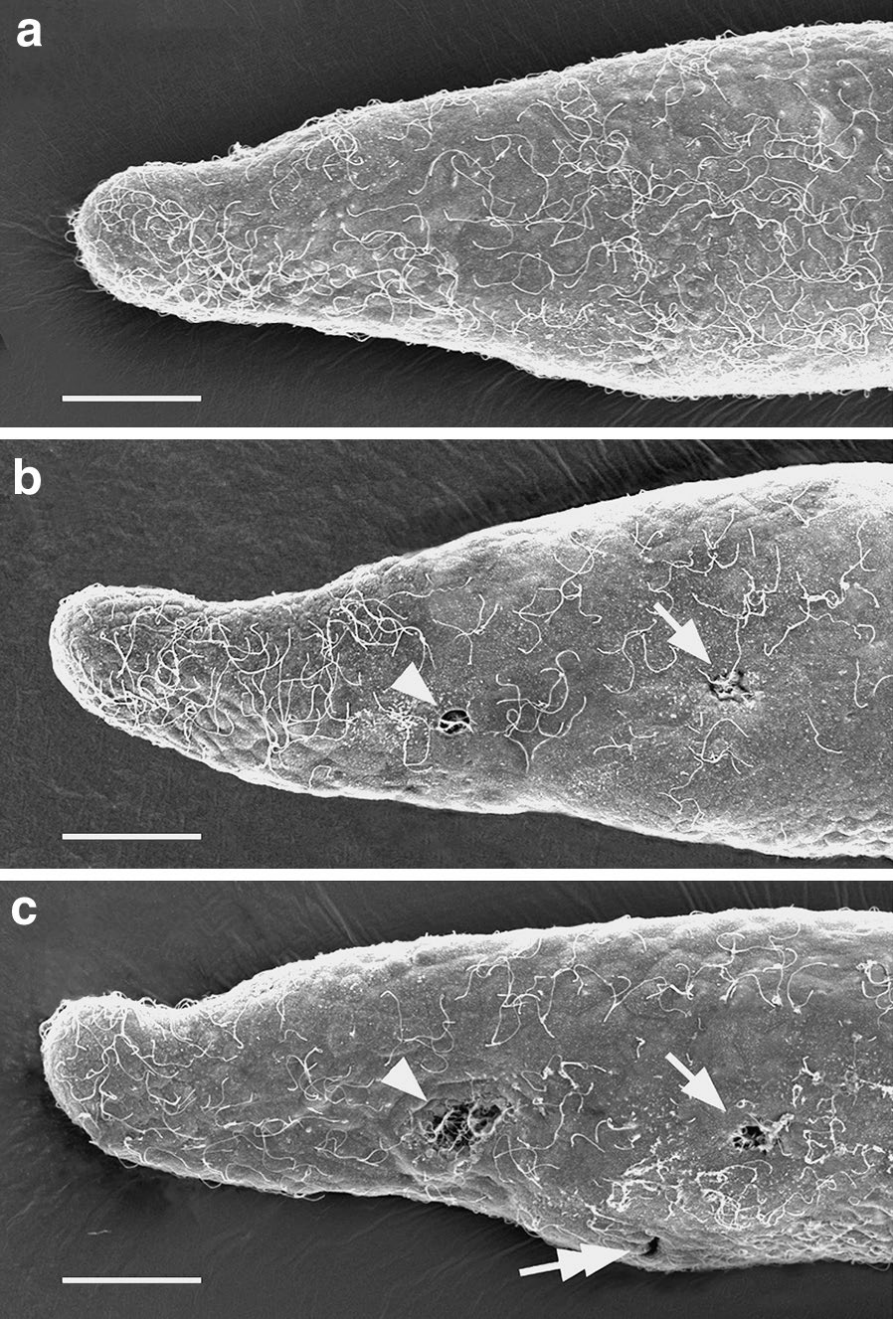
Conventional SEM series to determine the time of mouth opening. **a** 23-h larva; none the ten sampled had a mouth. **b** 24-h larva; one of two larvae out of ten that had open mouth (arrow) and preoral ciliated pit (arrowhead). **c** 25-h larva; all of the ten sampled had a mouth (arrow), an opening of the club-shaped gland (tandem arrow), and a preoral ciliated pit (arrowhead). All scale bars = 25 μm

Conventional SEM was used to accurately establish the time of mouth opening for larvae of the Florida amphioxus developing at 27 °C. In a sample of ten larvae at each time point, the mouth was present in none of them at 23 h of development, in two of them at 24 h, and in all of them at 25 h (Fig. 3a–c).

### Distinctive features of the first left somite

**Fig. 4:**
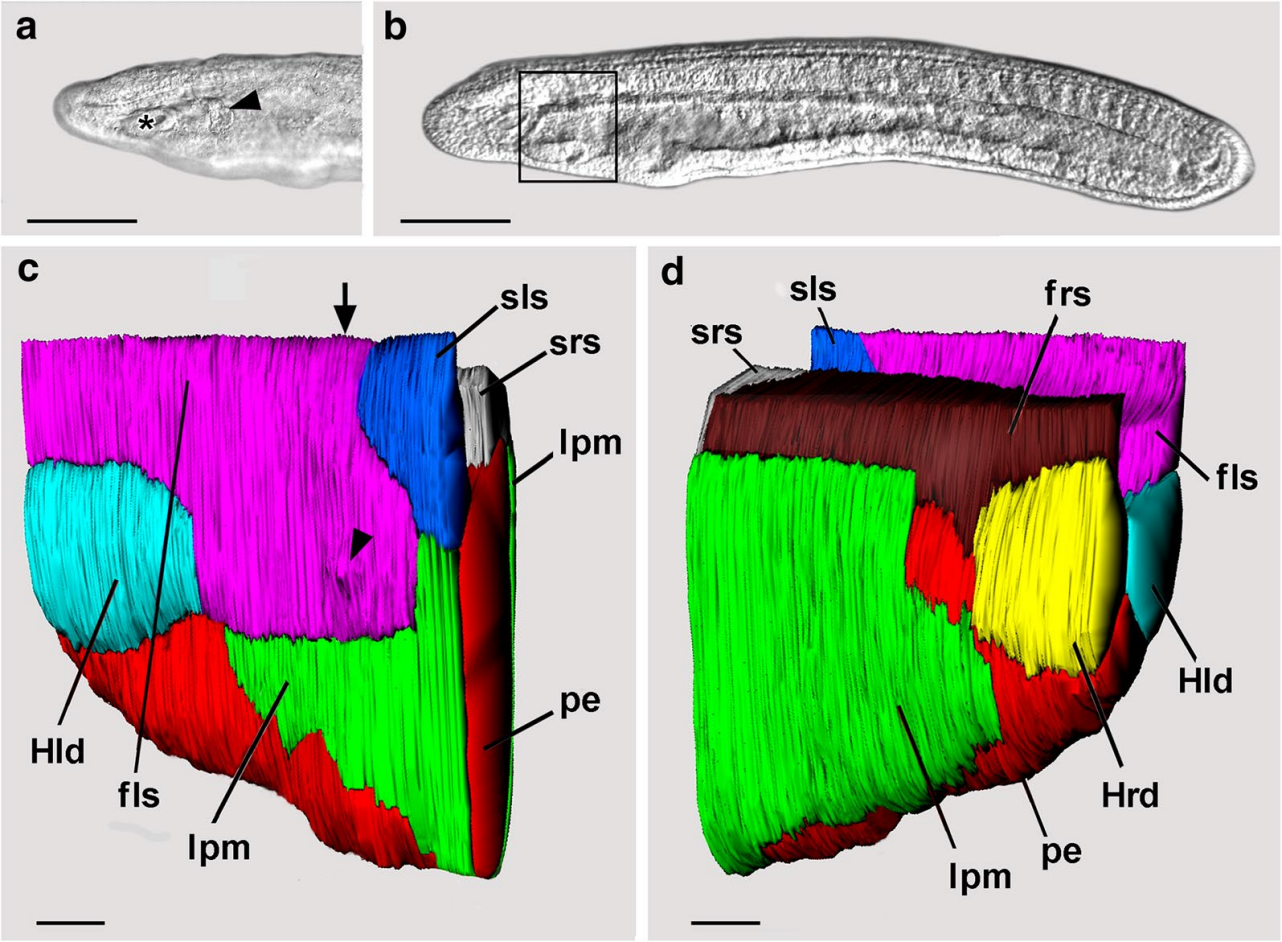
**a** DIC of anterior end of a living 22-h larva focused on the left surface, showing Hatschek’s left diverticulum (asterisk) and a population of compact mesoderm cells (arrowhead) comprising the posterior region of the first left somite. Scale bar = 100 μm. **b** DIC of a living 23-h larva with the midsagittal plane in focus. The boxed region indicates where SBSEM series were reconstructed in 3-D in **c** and **d**. Scale bar = 100 μm. **c** SBSEM of 23-h larva; left side view of a 3-D reconstruction; ectoderm is not shown. The arrowhead points to a small mesodermal protuberance showing where the mouth will later form. The arrow is explained in the following figure caption. Scale bar = 10 μm. **d** Right side view of a 3-D reconstruction. Scale bar = 10 μm. fls, first left somite; frs, first right somite; Hld, Hatschek’s left diverticulum; Hrd, Hatschek’s right diverticulum; lpm, lateral plate mesoderm; pe, pharyngeal endoderm; sls, second left somite; srs, second right somite

A few hours before the mouth opens, the first left somite contrasts with all the other somites (including its antimere on the right side) in two ways—first, it extends much farther ventrally and, second, its posterior wall comprises a population of compact mesoderm cells (diagrammed by coarse stippling in Fig. 1a). By the stage of the 22-h larvae, these clustered mesoderm cells are visible by DIC as a plaque just beneath the surface of the left side of the head (Fig. 4a, arrowhead).

**Fig. 5:**
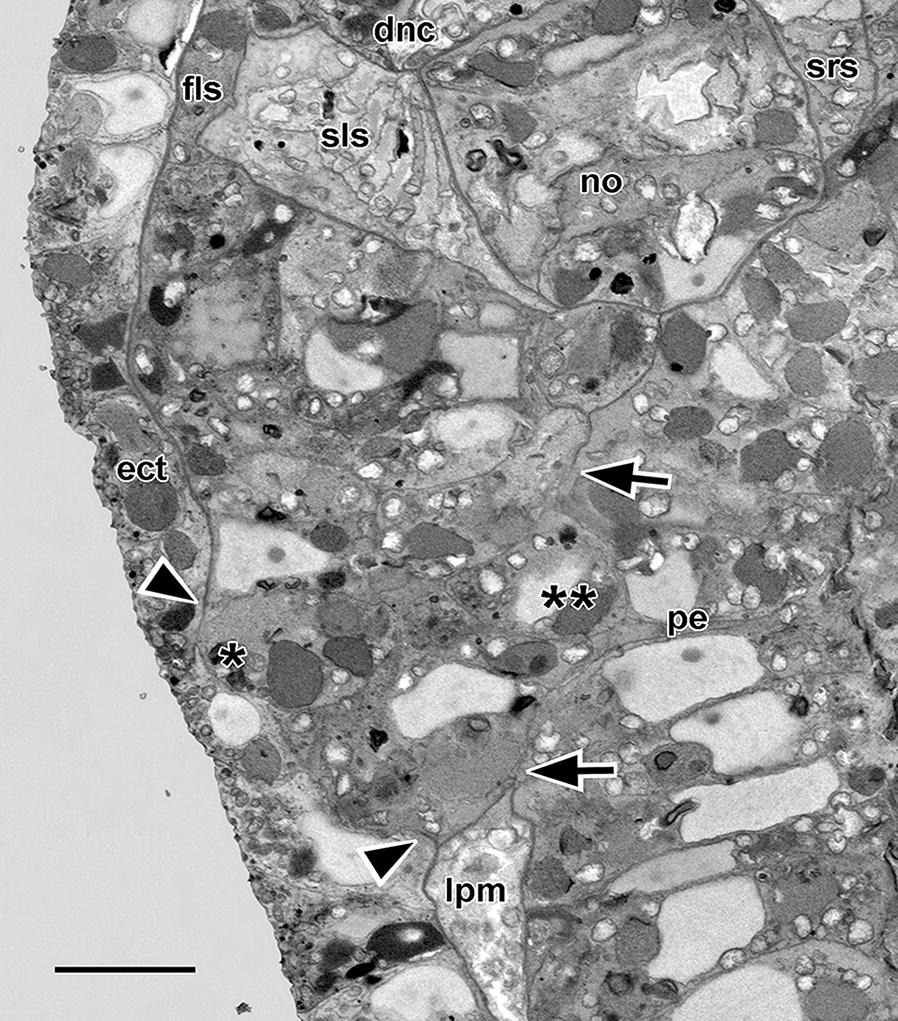
23-h larva. Single SBSEM scan through the arrowed level in Fig. 4c. The compact mesoderm cells of the first left somite bulge laterally (single asterisk) and medially (twin asterisk) toward the ectoderm and pharyngeal endoderm, respectively. For basal laminae, the arrowheads show the dorsoventral limits of the lateral window, and the arrowheads show the dorsoventral limits of the medial window. dnc, dorsal nerve cord; ect, ectoderm; fls, first left somite; lpm, lateral plate mesoderm; no, notochord; pe, pharyngeal endoderm; sls, second left somite; srs, second right somite. Scale bar = 5 μm

At the fine structural level (Fig. 5), these compact mesoderm cells lack cytoplasmic processes and include a nucleus with small nucleolus. Their cytoplasm contains free ribosomes, sparse profiles of endoplasmic reticulum, mitochondria, a Golgi complex, and yolk granules in various stages of dissolution; however, there was no evidence of microfilaments, microtubules, or specialized zones of cell-to-cell contact. The cells are probably not ciliated at this stage (however, cilia are inconspicuous in SBSEM scans and some might have been overlooked). It was certain, however, that none of the cells bore the flagellar/microvillar processes characterizing definitive nephridial cells (the cyrtopodocytes).

### 23-h larva, SBSEM

**Fig. 6:**
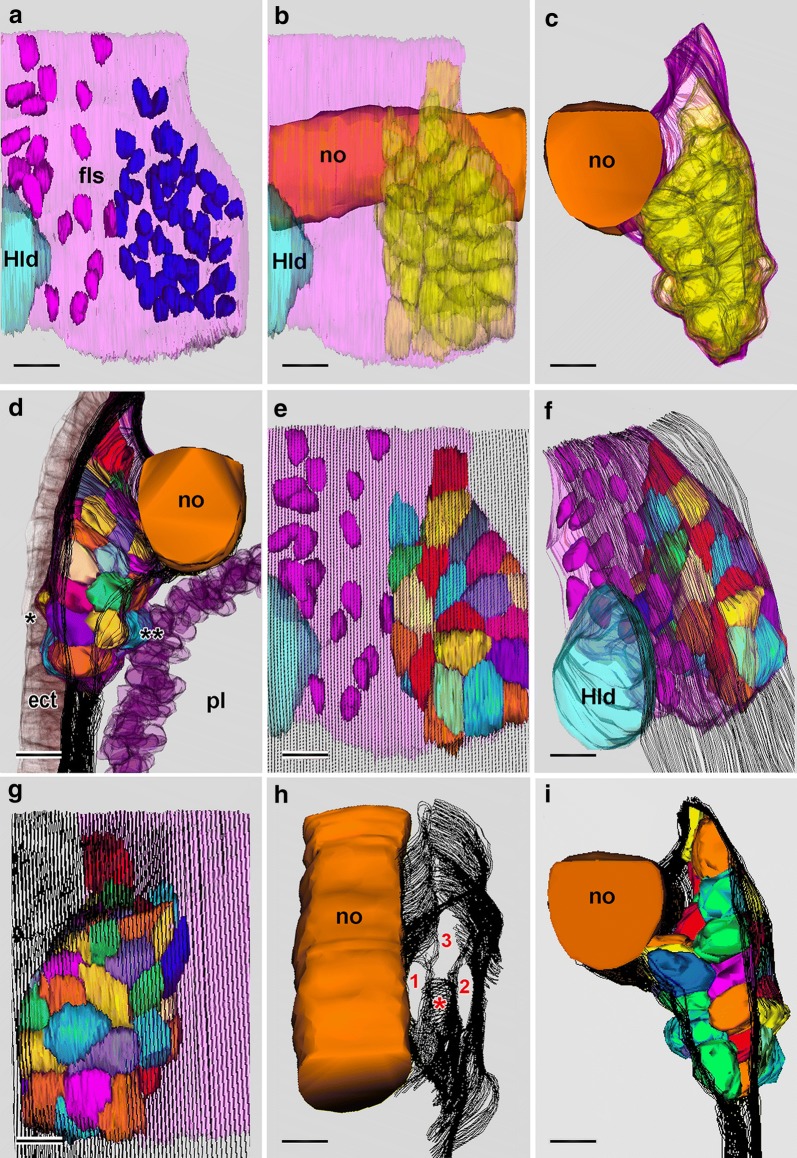
23-h larva. 3-D reconstructions based on SBSEM sequences. **a** Left side view of the posterior part of the first left somite (light pink) and part of Hatschek’s left diverticulum (light blue). Nuclei of the more anterior mesoderm cells are dark pink, and nuclei of the 48 compact mesoderm cells making up the posterior wall of the first left somite are dark blue. **b** Viewpoint of **a** with notochord orange and the first left somite light pink; the compact mesoderm cells of the posterior wall of the somite are shown in semitransparent yellow. **c** Similar to **b**, but turned to be viewed from the anterior. **d** View from posterior showing the compact mesoderm cells (in various colors) of the posterior wall of the first left somite; the notochord is orange, the nuclei of the endoderm cells are semitransparent purple, and the ectoderm cells (semitransparent brown) are locally compressed (asterisk) by the lateral protrusion of a few of the mesoderm cells; basal laminae are indicated by the parallel black traces. **e** View comparable to **a**, showing the nuclei of the more anterior mesoderm cells in dark pink, and the compact cells of the posterior wall in various colors; the basiepithelial basal lamina is indicated by the parallel black traces; a basal lamina-free area (the lateral window) is visible near the lower right. **f** The reconstruction in Fig. 5e turned to be viewed from the anterior left. **g** The compact mesoderm cells comprising the posterior wall of the first left somite viewed from the medial side; the subendodermal basal lamina (parallel black traces) is absent from an area (the medial window) near the bottom left. **h** The notochord and basal laminae (parallel black traces) of the left side from a dorso-anterior vantage point. The basal lamina ventral to the compact mesoderm cells of the first left somite is indicated by an asterisk; 1 and 2 are, respectively, the medial and lateral windows in the basal laminae, while 3 indicates the posterior communication of the first left somite with the lateral plate mesoderm. **i** Anterior view comparable to Fig. 5c; notochord is orange; compact mesoderm cells of the posterior wall of the first left somite in various colors, and basal lamina indicated by parallel black traces. All scale bars = 5 μm. ect, ectoderm; fls, first left somite, Hld, Hatschek’s left diverticulum, no, notochord; pl, pharyngeal lumen

In larvae less than 23 h old, no technique used here, including SBSEM (data not shown) could demonstrate exactly where the mouth would penetrate. By the stage of the 23-h larva, however, the site where the mouth will later open is indicated by a slight lateral protrusion the compact mesoderm cells (Fig. 4c, arrowhead) comprising the wall of the first left somite. In SBSEM reconstructions, 48 of these cells are present (Fig. 6a–g, i). The mesodermal protrusion is associated with a localized disappearance of the subectodermal basal lamina (Figs. 5 and 6e, f, h, i). The protrusion of mesoderm cells through this lateral window in the basal lamina somewhat compresses the neighboring ectoderm cells (indicated by the single asterisks in Figs. 5 and 6d). On the medial side of the cluster of compact mesoderm cells at this stage, a few cells (twin asterisks in Figs. 5 and 6d) bulge toward the pharyngeal endoderm. This medial protrusion is associated with a localized disappearance of the subendodermal basal lamina, which opens up a medial window in the lamina (Figs. 5 and 6g, h). In the posterior region of the first left somite, the cluster of compact mesoderm cells is separated ventrally from the underlying lateral plate mesoderm by a basal lamina (Fig. 6h). However, the mesoderm cells are not separated by basal laminae posteriorly from the lateral plate mesoderm of the second left somite (Fig. 6d, h) or anteriorly from the rest of the first left somite (Fig. 6i).

### 24-h larva, SBSEM

**Fig. 7:**
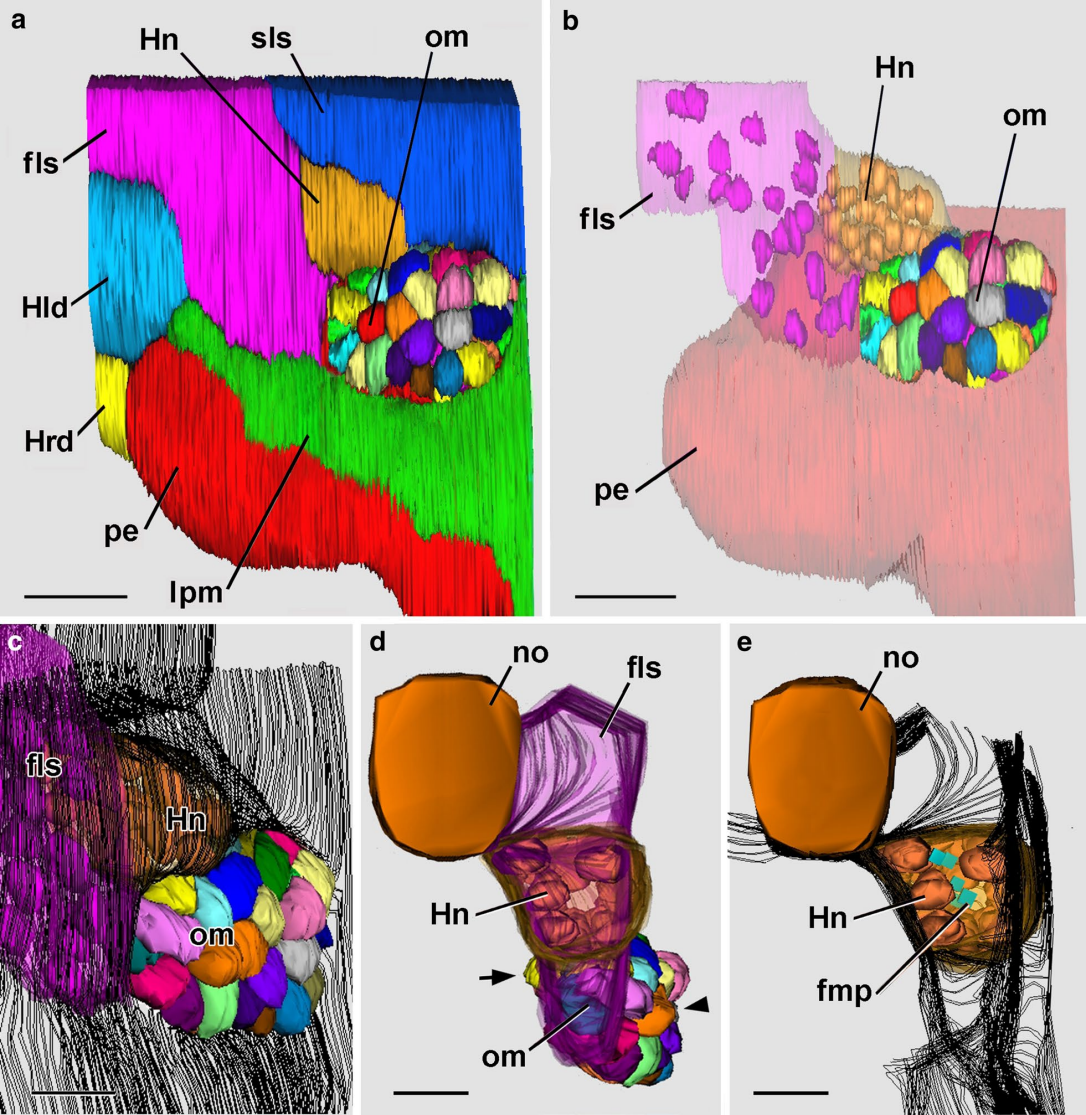
24-h larva. 3-D reconstructions based on SBSEM sequences. **a** Left side view of a reconstruction of a region similar to Fig. 4c; individual cells are depicted for the oral mesoderm. Scale bar = 10 μm. **b** Same view as **a**, showing cells for the oral mesoderm; the nuclei of Hatschek’s nephridium and the more anterior mesoderm cells of the first left somite are show, respectively, in gold and pink. Scale bar = 10 μm. **c** Oblique view of the right anterior side of the larva showing nuclei for the first left somite and for Hatschek’s nephridium as well as the cells of the oral mesoderm, which tends to bulge outward through the lateral window in the subectodermal basal lamina (indicted by the parallel black traces). Scale bar = 5 μm. **d** Anterior view including nuclei of Hatschek’s nephridium (gold); the cells of the oral mesoderm (various colors) bulge laterally (arrowhead) and medially (arrow) in regions where the basal lamina (nor shown) has disappeared. Scale bar = 5 μm. **e** Anterior view comparable to **d**, but showing flagellar/microvillar processes (blue rectangles) and nuclei of the Hatschek’s nephridium (gold); basal laminae are indicated by the parallel black traces. Scale bar = 5 μm. fls, first left somite; fmp, flagellar/microvillar process; Hld, Hatschek’s left diverticulum; Hn, Hatschek’s nephridium; Hrd, Hatschek’s left diverticulum; lpm, lateral plate mesoderm; pe, pharyngeal endoderm; om, oral mesoderm; sls, second left somite

**Fig. 8:**
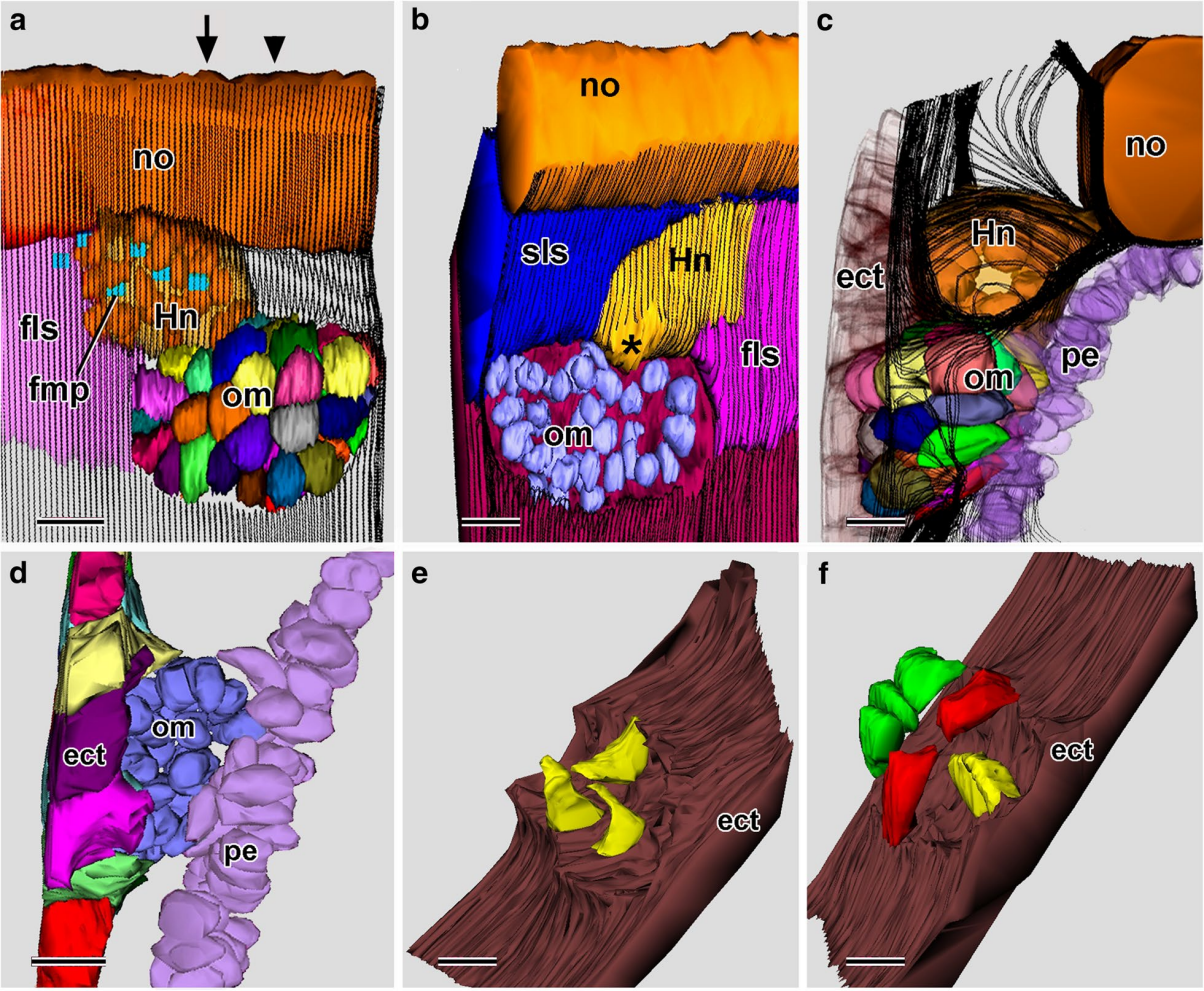
24-h larva. 3-D reconstructions based on SBSEM sequences. **a** Left-side structures from lateral viewpoint. The subectodermal basal lamina (indicated by parallel black traces) is interrupted (as a lateral window) over the oral mesoderm cells. Within Hatschek’s nephridium (rendered semitransparent) the cell nuclei (gold) are indicated as are several flagellar/microvillar processes (blue). The arrow and arrowhead are explained in the caption for Fig. 9. **b** Left-side structures from a medial viewpoint. The nuclei (blue) of the oral mesoderm cells are visible through a medial window in the subendodermal basal lamina. The asterisk marks an interruption in the basal lamina where Hatschek’s nephridium opens into the pharyngeal lumen. **c** View of left-side structures from the posterior showing that a basal lamina (parallel black traces) bounds Hatschek’s nephridium posteriorly but does not intervene between the oral mesoderm cells and the lateral plate mesoderm (toward viewer). **d** Similar view to **c** showing ectoderm cells (various colors), nuclei (blue) of the oral mesoderm, and nuclei (purple) of the pharyngeal endoderm. **e** Inner surface of the ectoderm (brown) viewed from anteriorly; dorsal and ventral are toward the upper right and lower left, respectively; three selected lateral cells of the oral mesoderm are shown in yellow. **f** Inner surface of the ectoderm (brown) viewed from posteriorly; dorsal and ventral are toward the upper right and lower left, respectively; for selected oral mesoderm cells, a lateral cell is yellow, a dorsal and a ventral cell are red, and three medial cells are green. ect, ectoderm; fls, first left somite; fmp, flagellar/microvillar process; Hld, Hatschek’s left diverticulum; Hn, Hatschek’s nephridium; no, notochord; pe, pharyngeal endoderm; om, oral mesoderm; sls, second left somite. All scale bars = 5 μm

In a 24-h larva without an open mouth, the cluster of compact mesoderm cells has clearly split into a dorsal population of cells comprising Hatschek’s nephridium and a ventral population of cells comprising what will be called here the “oral mesoderm” (Figs. 7a–d and 8a–c). This general term is introduced in preference to “mesovesicle” of Ref. [1], which is apt only during a very short period of development. There were 24 nephridial cells and 48 oral mesoderm cells in the 24-h larva, together representing an increase in cell number of nearly 30% from the 48 mesoderm cells at 23-h. Presumably, the increment resulted from cell division, which has previously been demonstrated in amphioxus larvae by labeling with bromodeoxyuridine [13]. Surprisingly, however, SBSEM images in the present study showed no mitotic figures (possibly an artifact due to SBSEM fixation).

**Fig. 9:**
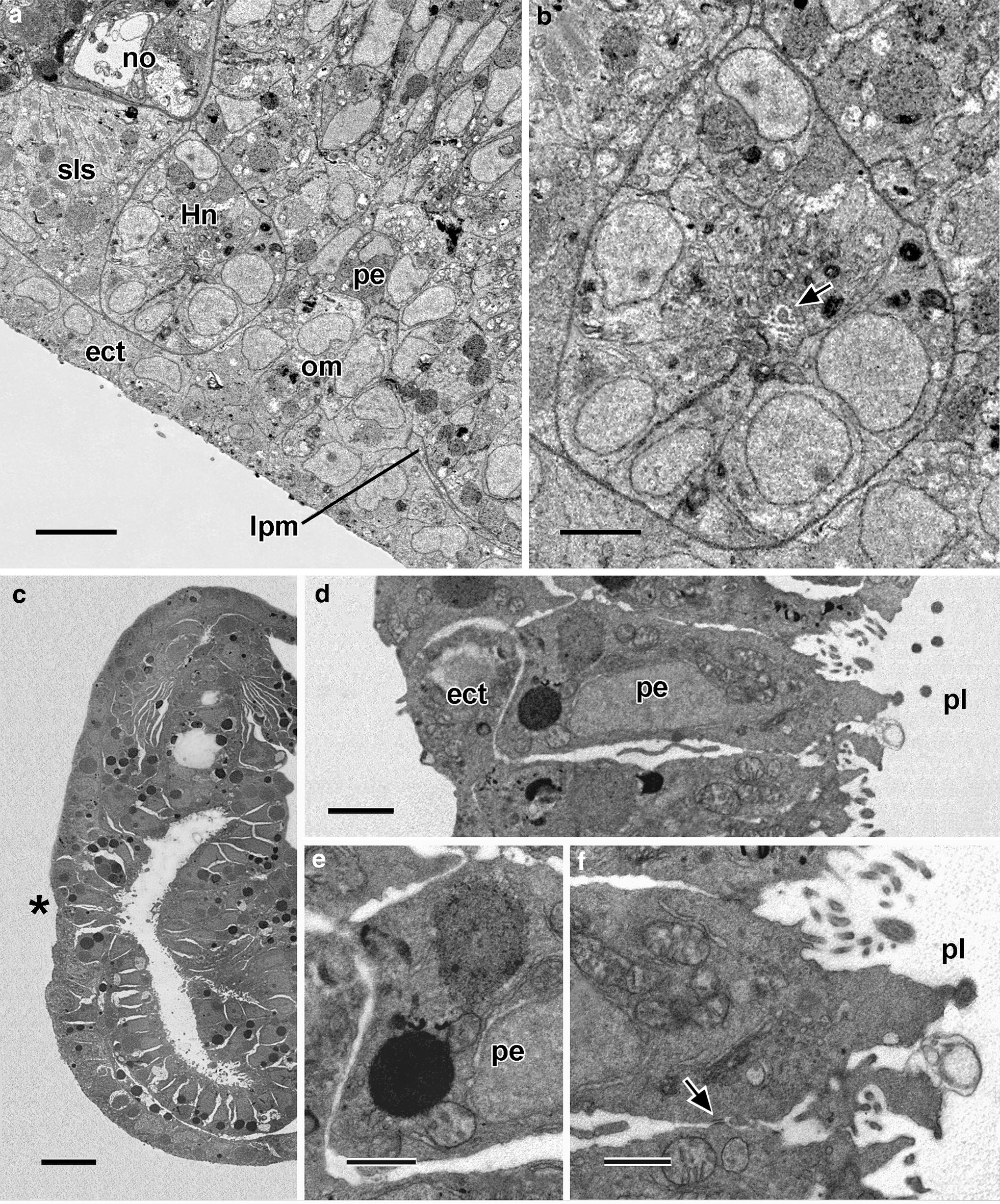
24-h larva. **a** Single SBSEM scan through the level indicated by the arrow in Fig. 8a showing structures on the left side. Scale bar = 5 μm. **b** Enlargement of **a** showing Hatschek’s nephridium, including a flagellar/microvillar process (arrow). Scale bar = 2 μm. **c** 24.5-h larva. Conventional TEM cross section at the level of the arrowhead in Fig. 8a. The asterisk indicates the region of body wall where pharyngeal endoderm cells are contacting the ectoderm. Scale bar = 10 μm. **d** Enlargement of the cells nearest the asterisk in **c**; pharyngeal endoderm cell (toward left) extends basally to contact an ectoderm cell (toward left). Scale bar = 2 μm. **e** The basal region of pharyngeal endoderm cell in **d** associated with an ectoderm cell at the left. Scale bar = 1 μm. **f** The apical region of pharyngeal endoderm cell in **d** associated with an adjacent endoderm cell by an inconspicuous intermediate junction (arrow). Scale bar = 1 μm. ect, ectoderm; Hn, Hatschek’s nephridium; lpm, lateral plate mesoderm; no, notochord; om, oral mesoderm; pe, pharyngeal endoderm; om, oral mesoderm; sls, second left somite

At 24 h, the forming Hatschek’s nephridium is growing directly posteriorly from the first left somite and is not then, or at any time in development, oriented approximately vertically as sometimes illustrated [14, 15]. At 24 h, the nephridium is open anteriorly to the rest of the first left somite (Fig. 7d, e) but separated by basal laminae from other neighboring structures (Figs. 7c and 8a–c) except at the place (Fig. 8b, asterisk) where the nephridium debouches into the pharyngeal lumen. Flagellar/microvillar processes, characteristic of cyrtopodocytes [4], were detected on five of the 24 nephridial cells (Figs. 7e, 8a, 9a, b). An additional flagellar/microvillar process was found on one cell located anterior to the nephridium (Fig. 8a); this may have resulted from the somewhat subjective placement of the boundary between the nephridium (with relatively densely packed nuclei) and the rest of the first left somite (with less densely packed nuclei). The advent of cyrtopodocytes took place somewhat later in the present study of the Florida amphioxus than in the European amphioxus [14], evidently due to a species difference in developmental rate.

In comparison with the nephridium, the cluster of oral mesoderm cells projects more posteriorly. Laterally and medially, basal laminae are absent, leaving windows where the oral mesoderm cells contact, respectively, the ectoderm and pharyngeal endoderm (Figs. 7c, 8a, b, 9a). Anteriorly, the oral mesoderm cells have become separated by a basal lamina from the rest of the first left somite (Fig. 8b), although posteriorly, they are still contiguous with neighboring cells of the lateral plate mesoderm of the second left somite (Fig. 8c, toward bottom left).

At 24 h, the cells of the oral mesoderm are transiently organized into a vesicular arrangement, as previously noticed in Ref. [1]. The more lateral cells are somewhat pear shaped (Fig. 8e), the dorsal and ventral ones are elongate (red in Fig. 8f), and the medial ones are more compact (green in Fig. 8f). Neither specialized cell–cell associations nor apical cilia were detected (although the apparent absence of the latter might have been a SBSEM fixation artifact). Importantly, at the time of the 24-h sample, the pharyngeal endoderm and ectoderm cells were completely separated by the intervening cluster of oral mesoderm cells.

### 24.5-h larva, conventional TEM

The cellular events associated with the opening of the mouth are very rapid, evidently on a time scale of only tens of minutes. In the present study, the number of SBSEM samples was limited, and none fell within this short temporal window. However, several of the conventional TEM samples fixed at 24.5 h demonstrated a stage in which the endoderm cells were displaced laterally to contact ectoderm cells directly, without any intervening oral mesoderm in the region where the mouth would soon penetrate (Fig. 9c–f). Although cells of the oral mesoderm were doubtlessly still present, they could not be unequivocally identified in the TEM sections, which were not collected in sufficiently long series to permit serial reconstructions. In addition, TEM only rarely demonstrated apoptotic cells in any larval tissues around the time of mouth penetration. The rarity of apoptosis in pre-metamorphic larvae of amphioxus is in agreement with TUNEL assays [16 and my unpublished data].

### 25-h larva, SBSEM

**Fig. 10:**
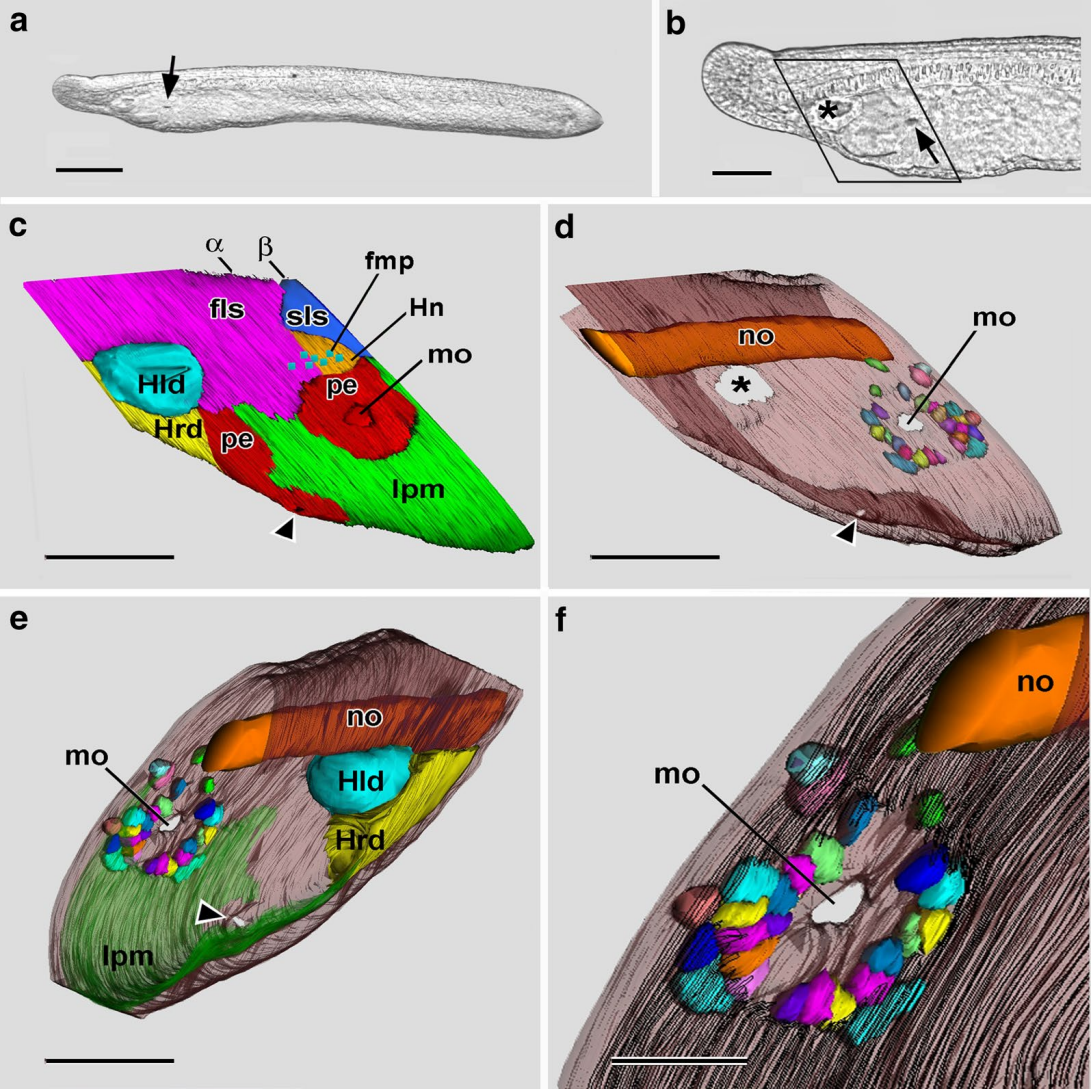
25-h larva. **a** DIC of living 25-h larva with recently opened mouth (arrow). Scale bar = 100 μm. **b** Enlargement of anterior end of **a**; asterisk and arrow indicate, respectively, Hatschek’s left diverticulum and mouth. The rhombus indicates the rombohedral volume where SBSEM series were reconstructed in 3-D in **c**–**e**. Scale bar = 50 μm. **c** Left side view of a 3-D reconstruction. The ectoderm is not shown. The arrowhead points to the external opening of the club-shaped gland; *α* and *β* indicate, respectively, the oblique planes of the SBSEM scans in Fig. 11a, b. Scale bar = 50 μm. **d** Same reconstruction as **d**, but showing the notochord and the semitransparent ectoderm, which is discontinuous over Hatschek’s left diverticulum (asterisk), opening of the club-shaped gland (arrowhead), and mouth (mo); the last is encircled by oral mesoderm cells (depicted in various colors). Scale bar = 50 μm. **e** Reconstruction like **d**, but turned to show left-side structures from a medio-posterior viewpoint. Scale bar = 50 μm. **f** enlargement of the oral region of **e**, with the subendodermal basal lamina indicated by parallel black traces. Scale bar = 20 μm. ect, ectoderm; fls, first left somite; fmp, flagellar/microvillar process; Hld, Hatschek’s left diverticulum; Hn, Hatschek’s nephridium; Hrd, Hatschek’s right diverticulum (often termed the rostral coelom by this stage of development); lpm, lateral plate mesoderm; mo, mouth; no, notochord; pe, pharyngeal endoderm; pl, pharyngeal lumen; om, oral mesoderm; sls, second left somite

**Fig. 11:**
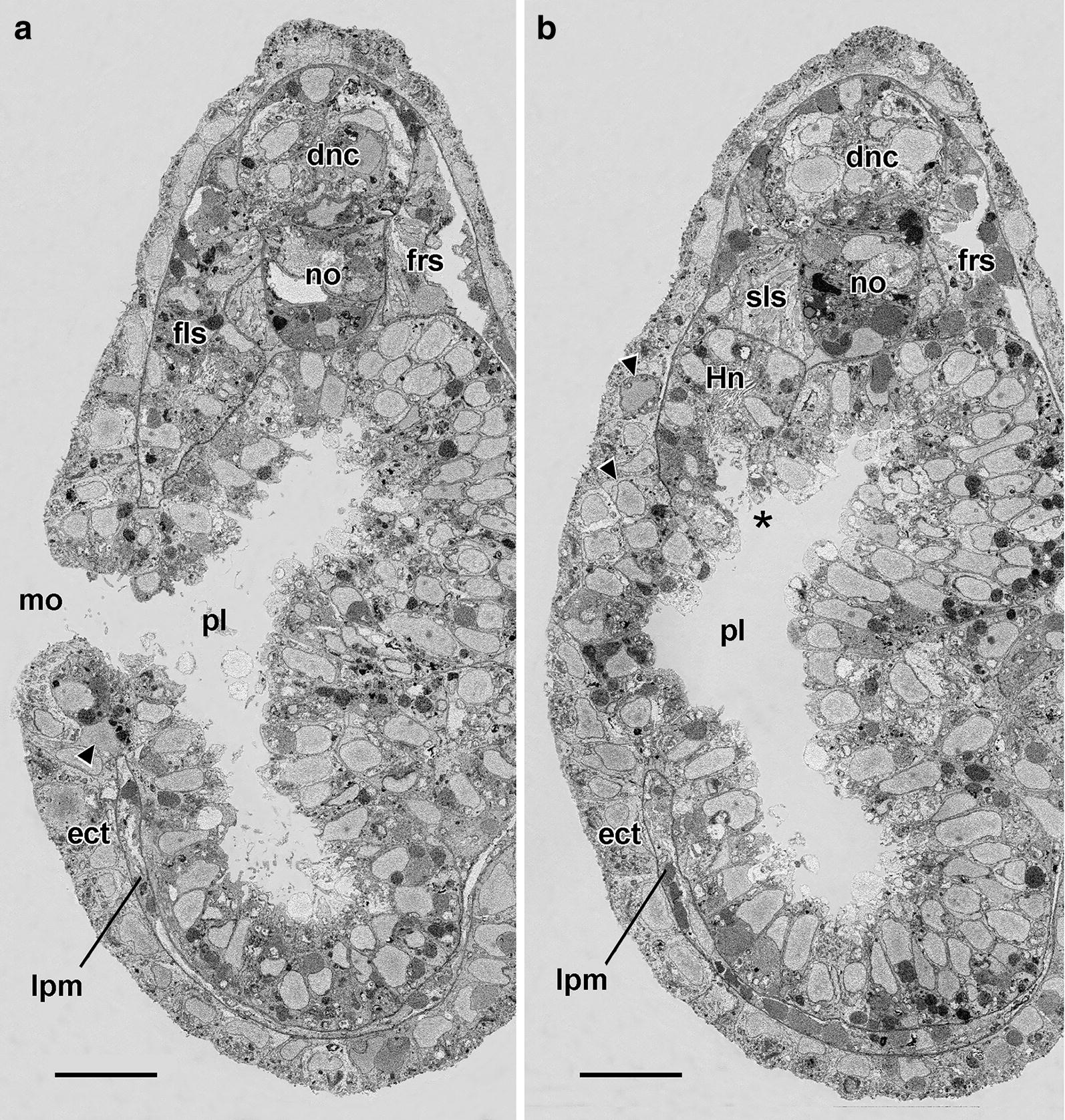
25-h larva. **a** Single SBSEM scan through the oblique plane indicated by *α* in Fig. 10c. The arrowhead points to an oral mesoderm cell. Scale bar = 10 μm. **b** Single SBSEM scan through the oblique plane indicated by *β* in Fig. 10c. The arrowheads point to oral mesoderm cells, and the asterisk marks the opening of Hatschek’s nephridium into the pharyngeal lumen. Scale bar = 10 μm. dnc, dorsal nerve cord; ect, ectoderm; fls, first left somite; frs, first right somite; Hn, Hatschek’s nephridium; lpm, lateral plate mesoderm; mo, mouth; no, notochord; pl, pharyngeal lumen; sls, second left somite

In the 25-h larvae, the mouth had penetrated (Fig. 10a, b). The 25-h SBSEM specimen (Fig. 10c) had been embedded with its anterior end slanted dorsally. This meant that the reconstructed tissue volume was rhombohedral (diagrammed in Fig. 10b). Although this resulted in some minimal distortion (e.g., in Hatschek’s nephridium, Fig. 3c), the 3-D reconstructions were still informative. The most striking finding was that the recently penetrated mouth was surrounded by a circle of oral mesoderm cells (Figs. 10d–f and 11a, b). These cells were probably myoblasts destined to give rise to the perioral musculature later in development—as previously suggested by Lacalli [17]. The oral mesoderm cells closest to the mouth were sandwiched between the pharyngeal endoderm and the ectoderm (Figs. 10f and 11a, arrowhead), while those farther away were located between the ectoderm and the subectodermal basal lamina (Figs. 10f and 11b, arrowheads). It is not known whether the oral mesoderm cells encircling the new mouth were passively pushed aside by the interaction of the endoderm and ectoderm or actively withdrew or both. Whereas there had been 48 mesoderm cells in the 24-h larva, only 29 were detected in the 25-h sample. The possible fates of the missing cells are considered in the “Discussion” section.

## Discussion

### Apparent decline in number of oral mesoderm cells as the mouth opens

As mentioned, the number of oral mesoderm cells fell from 48 to 29 when the mouth penetrated. The decline was not likely due to apoptosis, which was only rarely detected in the tissues of the early larvae. This raises the obvious question of what happened to the missing 19 cells (and presumably somewhat more than that if one assumes that some cell division was taking place). It remains possible that Ref. [1] was correct in the sense that some of the oral mesoderm cells are added to the rim of the nascent mouth. On the other hand, the present study demonstrated that at least some of the ectoderm and endoderm cells can contact each other directly while the mouth is opening (Fig. 9c–f) without the interposition of an obvious sleeve of oral mesoderm cells.

There are, however, alternative explanations for the smaller number of oral mesoderm cells detected after mouth penetration. The first could be that the morphogenetic interaction of the endoderm and ectoderm cells (e.g., Fig. 9d) is disruptive enough to force some of the cells in question out into the surrounding environment. The second reason for the discrepancy could simply be statistical. A single specimen shortly before mouth penetration was compared with a single specimen just after the mouth had formed; thus the dispersion of data around the mean is not known for either sample. It is possible that the number of oral mesoderm cells around the time of mouth opening varies enough from one larva to the next to account for the difference in cell number reported here. In any case, the results in the present paper, like those in Ref. [1], are based on static histological data, and dynamic cell tracer experiments would be required to provide a more definitive answer about the fate of the missing oral mesoderm cells.

In contrast to the uncertainty surrounding the vanished oral mesoderm cells, something more definite can be said about those that remain after the mouth opens. As already mentioned, it is likely that such cells are precursors of the perioral musculature. This possibility is strengthened by some of the molecular data in Ref. [1], which showed that the oral mesoderm cells expressed genes involved in myogenesis (*Pax3/7* and *mef2*), as would be expected if the cells there were myoblasts destined to differentiate into muscles associated with the mouth.

### The coelomoduct mouth scenario in a broader context

For many years, there were two major schools of thought about amphioxus mouth evolution. The first held that all chordate mouths are homologous, although there was much debate about what historical or mechanical factors might account for the divergent locations of oral openings from one major group to the next [18]. The second main school of thought held that the cephalochordate mouth is homologous, not to mouths in other chordates, but to a vertebrate gill slit. In the words of van Wijhe [19], “Amphioxus cannot hear; he eats however with the left ear, and has consequently lost the mouth.” Proponents of a gill slit mouth have put forward diverse ideas about what constituted the original cephalochordate mouth before its function was preempted.

Reference [1] recently taken a third position in the argument about amphioxus mouth evolution by contending that amphioxus inherited an excretory coelomoduct from ambulacrarian (echinoderm-like or hemichordate-like) ancestors and began eating with it. The coelomoduct mouth scenario was based in part on morphological observations—which the present study could not conclusively refute, but does not necessarily support—and in part on molecular data, including developmental expression patterns for several genes (some functionally manipulated in down-regulation experiments) as well as immunochemical observations. In the opinion of Ref. [20], the most relevant gene for supporting the coelomoduct mouth scenario was *BMP2/4*, which was purportedly expressed in intimate association with the forming mouth. Since then, however, the mouth-associated expression of *BMP2/4* claimed in Ref. [1] has been shown to have been a mistaken observation [21].

Reference [1] claimed that *Lim1* expression is “nephridium specific,” but this is stretching things because the gene is also expressed in several other tissues during amphioxus development [22]. In addition, Ref. [1] proposed that POU-IV expression, originally demonstrated in larval amphioxus by reference [23], supported, in some unspecified way, the coelomoduct mouth hypothesis. Alternatively, however, POU-IV expression inhibits synthesis of plakoglobin [24], a protein involved in cell–cell association, and the strong expression of the gene in the oral mesoderm cells could conceivably be involved in their dispersion during mouth formation and have nothing to do with their proposed addition to the rim of the forming mouth. Finally, the expression domains of several other genes in Ref. [1] (namely, *Dickkopf*, *Frizzled*-*related*, and *Pax2/5/8*) as well as the antilaminin staining correlate well with the disappearance and re-synthesis of basal laminae in the oral region, but say nothing about the addition of mesoderm cells to the nascent mouth rim.

### Earlier conceptions of larval mouth penetration in amphioxus

The population of compact cells in the posterior region of the first left somite (Fig. 4a, arrowhead) was seen a century ago by Smith and Newth [25]. From examining histological sections, they described what they called a “superficial aggregation of cells” on the left side of the larval head. They claimed that the cells in question were the rudiment of Hatschek’s nephridium. In addition, they tentatively floated the idea that their cell aggregation was ectodermal and not mesodermal in origin. Although this conception of nephrogenesis in amphioxus was incorrect, it was welcomed by Goodrich [15, 26], who was anxious for the nephridium of amphioxus to be ectodermal because its cells were characterized by flagellar/microvillar processes that reminded him of the ectodermal protonephridia of some protostome invertebrates [4]. In addition, Smith and Newth [25] followed earlier anatomists [19, 27, 28] in proposing the oversimplified notion that the amphioxus ectoderm simply underwent “fusion with the gut, to form the mouth.” It is still possible that that amphioxus mouth penetration results from fusion of ectoderm and endoderm—and thus resembles mouth and gill slit penetration in other chordates [29–32]. However, the participation of mesoderm cells in the penetration of the larval mouth of amphioxus has not yet been ruled out conclusively. Reference [1] deserved credit for calling attention to the possible participation of mesoderm cells in the formation of the larval mouth of amphioxus.

### The utility and future of SBSEM as an embryological tool

Beyond accomplishing the immediate aims of this study (namely, describing nephrogenesis and mouth formation), SBSEM gave insights on additional subjects. For example, the more exact information on the topology of the various coelomic compartments does not support the proposal in [34] that there is a connection between Hatschek’s right diverticulum (= rostral coelom) and the more posterior coelomic cavities. The construction of 3-D models based on SBSEM also promises to answer some additional questions about amphioxus coeloms. For example: what is the ultimate fate of Hatschek’s right diverticulum (known later in development as the rostral coelom)? MacBride [35] claimed it ultimately vanishes, but Franz [36] contended that a trace of it persists just beneath the dorsal nerve cord. Also, are an upper lip coelom and a lower lip coelom cut off from the perivisceral coelom during amphioxus development, as claimed by Jefferies [37]?

It will be especially interesting to use SBSEM to determine how much Hatschek’s nephridium development and mouth penetration have in common with the formation of the branchial nephridia and pharyngeal gill slits. The development of these last two structures was described many years ago by light microscopists [38–41] who illustrated their findings with figures that were more diagrammatic than realistic and have been uncritically accepted since then [1]. To date, the only TEM study of larval gill slits in amphioxus larvae was chiefly concerned with the musculature [42], and SBSEM appears to be a promising tool for reinvestigating development of the branchial nephrogenesis and gill slit penetration in amphioxus larvae.

Until now, developmental biologists have chiefly used SBSEM to describe tissues (especially neural) dissected from relatively large embryos of vertebrates [10, 33]. However, the present study shows that the method can be applied effectively for describing large regions of intact tissues in the relatively small developmental stages of amphioxus. There is every reason to believe that SBSEM would be similarly useful for describing the embryos and larvae of other invertebrates where much structural complexity is packed into a relatively small volume. At present, however, it unlikely that SBSEM would be a fruitful approach for in situ descriptions of the relatively large developmental stages characteristic of some invertebrates and many vertebrates: The technique requires extensive infiltration of contrast-enhancing reagents into the tissues, so the larger the specimen, the greater the potential for inadequate contrast or poor fixation or both. Even so, it is possible that fixation methods will be refined and better instrumentation will be developed, making SBSEM more generally useful for the study of development.

## Conclusion

For early larvae of amphioxus, developmental changes are described at the tissue and cell level by serial blockface scanning electron microscopy (SBSEM). Special attention is given to the fate of the mesoderm cells at the posterior end of the first left somite. A dorsal group of these cells develops into the initial kidney (Hatschek’s nephridium), while a ventral group (termed there the oral mesoderm) becomes interposed between the ectoderm and endoderm in the region where the mouth will soon form. After mouth penetration, many of the oral mesoderm cells are still detectable and probably represent myoblasts destined to give rise to the perioral musculature. The present study found no morphological evidence that some of the other oral mesoderm cells become intercalated into the rim of the nascent mouth—such an addition is indispensable for the coelomoduct mouth scenario of Kaji et al. [1]. Even so, the developmental fate of the mesodermal cells in question could only be demonstrated conclusively by dynamic cell tracer studies. In sum, for now, there is no reason to prefer the coelomoduct mouth scenario to other ideas that have been proposed to explain the evolutionary history of the oral opening of amphioxus.
